# A rehabilitative nursing intervention for elderly victims of spousal emotional abuse and its impact on stress level, life satisfaction, and family functioning

**DOI:** 10.1186/s12912-025-04255-7

**Published:** 2026-02-03

**Authors:** Nabila Elsayed Sabola, Ahmed Hussien Ahmed Kotb, Hayam Labib Matter, Amal Yousef Abdelwahed, Olfat Abdulgafoor Gushgari, Fadiyah Abdullah Alshwail, Rowaedh Ahmed Bawaked, Marwa Abdelfattah Elsalamony, Rasha Kamal Sweelem, Arwa Abd El Salam Ashry, Ghada Mohammed Elhgry

**Affiliations:** 1 Department of Community Health Nursing, Faculty of Nursing, Menoufia University, Menoufia, Egypt; 2 Department of Nursing Administration, Faculty of Nursing, Fayoum University, Fayoum, Egypt; 3 Department of Geriatric Health Nursing, Faculty of Nursing, Menoufia University, Menoufia, Egypt; 4 Department of Public Health, College of Health Sciences, Saudi Electronic University, Dammam- Jeddah- Riyadh, Saudi Arabia; 5 Department of Psychiatric and Mental Health Nursing, College of Nursing, Taif University, Taif, Saudi Arabia; 6 Department of Psychiatric and Mental Health Nursing, College of Nursing, Northern Border University, Arar, Saudi Arabia; 7 Department of Community Health Nursing, Faculty of Nursing, Damanhour University, Beheira, Egypt

**Keywords:** Nursing rehabilitative intervention, Emotional abuse, Family functioning, Life satisfaction

## Abstract

**Background:**

Spousal emotional abuse is a serious form of maltreatment that is highly prevalent. It threatens the well-being of elderly couples and negatively impacts family functioning. Therefore, this study aimed to evaluate the impact of a rehabilitative nursing intervention for elderly victims of spousal emotional abuse on stress level, life satisfaction, and family functioning.

**Methods:**

A quasi-experimental research design was used in this study, which was conducted in Estabary and Ganzor villages in Menoufia Governorate, Egypt. A purposive sample of 50 elderly couples experiencing spousal emotional abuse (50 wives and 50 husbands) was recruited. Data were collected using four tools: the Multidimensional Emotional Abuse Questionnaire, the General Functioning 12-item subscale, the Satisfaction with Life Scale, and the Stress Scale.

**Results:**

The mean scores of the elderly couples for total family functioning and total life satisfaction significantly increased in the post-intervention phase (34.68 ± 5.15, 25.4 ± 3.5, respectively) compared to the pre-intervention phase (20.04 ± 4.08, 19.2 ± 3.6, respectively). Additionally, the mean scores for total emotional abuse and total stress significantly decreased in the post-intervention phase (85.2 ± 20.3, 6.7 ± 1.9, respectively) compared to the pre-intervention phase (117.7 ± 21.4, 16.2 ± 3.1, respectively).

**Conclusion:**

The rehabilitative nursing intervention for elderly victims of spousal emotional abuse was effective in reducing stress levels and improving life satisfaction and family functioning. Therefore, these interventions should be implemented with support from specialized professionals and institutions.

## Background

The marital relationship is an important source of well-being for older adults. The majority of a married adult’s daily time is spent with their spouse, and emotional abuse can affect people of all ages, occurring at any stage of life [1]. Emotional abuse is the most common form of intimate partner violence worldwide [2]. Studies show that the consequences of emotional abuse are more severe than those of physical abuse [3]. Marital emotional abuse has short-term effects on the victim, such as fear, helplessness, feelings of shame, mood swings, aches and pains, difficulty concentrating, and muscular tension. Long-term effects include insomnia, chronic pain, social isolation, loneliness, guilt, and stress [4].

Older adults are a demographic group at heightened risk for various vulnerabilities, including physiological changes, disability, social isolation, chronic illness, and cognitive impairment. These factors can make individuals more susceptible to experiencing new or escalating forms of violence, especially emotional abuse [5]. As a result, elderly individuals are more vulnerable to emotional abuse even within healthy relationships or long-term marriages lasting several decades [6]. In Egypt, the number of older adults is estimated to be 3.7 million men (approximately 6.9% of the total male population) and 3.2 million women (roughly 6.4% of the total female population), according to population projections for 2022 [7].

Emotional abuse among spouses is a pattern of behavior in which one partner uses non-physical actions to control, isolate, or demean the other, causing emotional harm. This can include insults, threats, controlling behavior, and attempts to isolate the victim from friends and family. The prevalence is high: one in six men and one in four women report having experienced emotional abuse from a spouse [1]. Nearly half of American men and women (48.4% and 48.8%, respectively) have been victims of emotional abuse in an intimate relationship [5]. Almost 12 million Americans report having experienced emotional abuse in a close relationship. It is also more prevalent than physical or sexual abuse [8]. A study conducted in Egypt on domestic violence found that 41.80% of the sample had experienced psychological violence or emotional abuse [9].

Emotional abuse between spouses is diagnosed when the relationship remains strained over an extended period of time [8, 10, 11]. An individual may be involved in an emotionally abusive marital relationship if their partner tries to control them through behaviors such as name-calling, constant insults or criticism, acting possessive or jealous, and lacking trust. Other forms of emotional abuse may include humiliating them in public or private, provoking them, damaging their property (e.g., tossing objects, punching walls, kicking doors), and then blaming them for the abuser’s aggressive actions [12].

People who experience emotional abuse may struggle with regulating their emotions or forming positive interpersonal connections later on. Emotional abuse is linked to negative psychological effects such as depression, persistent anxiety, stress, sleep difficulties, and decreased life satisfaction among older couples. It can also lead to various health conditions and complications, including chronic pain, headaches, irritable bowel syndrome, and post-traumatic stress disorder. Additionally, chronic illnesses like fibromyalgia and chronic fatigue syndrome may be exacerbated by emotional maltreatment [4].

Life satisfaction is a comprehensive assessment of one’s overall feelings and attitudes toward life. It is a person’s cognitive and introspective evaluation of how well they are doing and how fulfilled they feel [13]. Spouses who experience emotional abuse tend to have lower self-esteem, are more likely to experience a persistent low mood, require psychological support, and report lower life satisfaction [14].

Family functioning refers to a family’s ability to navigate daily life and cope with the problems and challenges that arise. Effective family functioning occurs when family members fulfill their respective roles, successfully complete practical tasks, and maintain relationships both within and outside the family context. A family with adequate levels of functioning is more likely to address critical situations with emotional stability and communicate effectively [15]. Stress refers not only to the psychological feeling of pressure or tension but also to the body’s physical and emotional response to that feeling. Spousal emotional abuse significantly impacts both family functioning and stress levels. Victims of emotional abuse often experience a decline in self-esteem, increased anxiety and depression, and difficulties in interpersonal relationships, all of which negatively affect the family dynamic. The abuse can also lead to heightened stress, impacting overall well-being and potentially creating a cycle of negativity within the family [16].

Despite the widespread prevalence of emotional abuse among elderly couples, evidence-based interventions to prevent and address this issue remain insufficient. Most interventions targeting psychological abuse and neglect have been implemented in the USA and primarily focused on specialized psychological support delivered by social workers, usually in healthcare facilities and community centers. However, there is limited research on the application of rehabilitative nursing interventions to manage emotional abuse. Nurses can play a critical role in addressing emotional abuse by identifying at-risk individuals, providing support, and advocating for their safety [11, 17]. Emotionally focused interventions for couples provide an opportunity for spouses to gain a better understanding of their relationship and emotionally empower them to address their issues. These interventions also help individuals develop skills for responding to difficult emotions and learn strategies for effectively regulating negative emotions [18].

## Aim of the study

**Fig. 1:**
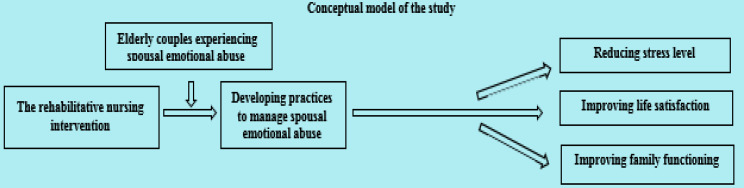
Conceptual model illustrating the relationships among the study variables

The present study aims to evaluate the impact of a rehabilitative nursing intervention for elderly victims of spousal emotional abuse on stress level, life satisfaction, and family functioning. The study hypothesizes that elderly victims of spousal emotional abuse who receive the rehabilitative nursing intervention will show a reduction in stress levels and an improvement in both life satisfaction and family functioning (Fig. 1).

## Methods

### Study design

A quasi-experimental research design (a pretest - posttest one group) was used in the current study.

### Study setting

This study was conducted in the Estabary and Ganzor villages, located in the Shebin El-Kom district of Menoufia Governorate, Egypt. Menoufia Governorate comprises ten districts, with the Shebin El-Kom district consisting of 36 villages. The population of Estabary is approximately 12,000 individuals, while Ganzor has a population of around 16,500 individuals.

### Study participants

**Fig. 2:**
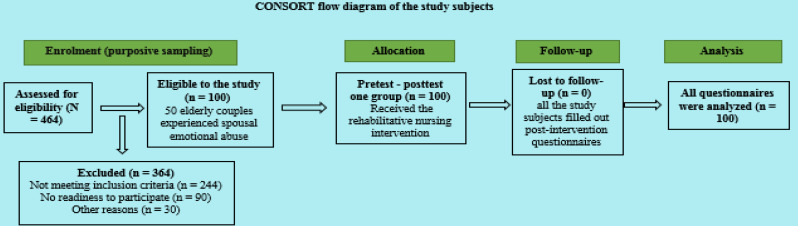
The CONSORT flow diagram illustrating the number of study subjects at each phase of the study

As shown in Fig. 2, a purposive sample of 50 elderly couples (50 wives and 50 husbands) experiencing spousal emotional abuse was recruited (*n* = 100) after being screened with the question: “Are you exposed to emotional abuse from your partner?” If the response was “yes” for one or both partners, they were considered to be experiencing emotional abuse. The inclusion criteria were elderly couples aged 60 years or older, with a marriage duration of at least five years, who had been facing emotional challenges in their relationship for more than one year, and with no history of psychiatric illness. The researchers excluded elderly couples who were illiterate or who had experienced other forms of abuse (physical, financial, or sexual) or were using antidepressants, anti-anxiety medications, or narcotic drugs. To avoid the potential selection bias, the authors clearly define objective inclusion and exclusion criteria in advance.

### Data collection tools

Data were collected using four tools: the Multidimensional Emotional Abuse Questionnaire, the Stress Scale, the Satisfaction with Life Scale, and the General Functioning 12-item subscale.

### Tool (I): Multidimensional Emotional Abuse Questionnaire (MMEAQ)

This tool was designed to assess the prevalence and various aspects of emotionally abusive behaviors among elderly couples experiencing spousal emotional abuse throughout the intervention phases. It consists of two parts:

#### Part 1

This part included a structured interview questionnaire to collect demographic data (gender, age, education level, etc.) and clinical/familial information (e.g., parental history of abuse, smoking, chronic illnesses, etc.).

**Part 2**: This part was developed by Murphy and Hoover [19] and modified by the authors through the addition of new expressions and translation. It consists of twenty-eight items, classified into four dimensions: restrictive engulfment, denigration, hostile withdrawal, and dominance/intimidation, with seven items in each dimension. Participants were asked to report how frequently they had experienced these abusive behaviors over the past year using a seven-point frequency scale (0 = never; 1 = once; 2 = twice; 3 = 3–5 times; 4 = 6–10 times; 5 = 11–20 times; 6 = more than 20 times). For each dimension of emotional abuse, as well as for total emotional abuse, the item scores were summed and then divided by the number of items, providing a mean score for each dimension and for total emotional abuse. Higher mean scores indicated a higher incidence of emotional abuse, and vice versa [20].

### Tool II: Stress Scale

The Stress Scale was designed to assess difficulty relaxing, nervous arousal, irritability, over-reactivity, impatience, and the tendency to become easily upset or agitated among the study participants. It is part of the Depression, Anxiety, and Stress Scale-21 Items (DASS-21) developed by Lovibond and Lovibond [21]. The authors modified the Stress Scale by adjusting the scoring system. This tool consists of seven items, each with a four-point Likert scale ranging from “not at all” (score = 0) to “mostly” (score = 3). The total score for participants ranged from 0 to 21. Higher scores indicated higher levels of stress, and vice versa [22].

### Tool III: Satisfaction with Life Scale (SWLS)

This tool was developed by Diener et al. [23] and modified by the authors through the addition of new expressions and translation to assess individuals’ self-reported satisfaction with life in general. It consists of five items, each rated on a seven-point Likert scale, ranging from “strongly disagree” (score = 1) to “strongly agree” (score = 7). Participants’ total scores ranged from 5 to 35. Higher scores indicated greater life satisfaction, and vice versa [24].

### Tool IV: General Functioning 12-item subscale (GF12)

The General Functioning 12-item subscale (GF12), a component of the McMaster Family Assessment Device (FAD), was validated by Cong et al. [25] as a separate measure with strong psychometric properties for assessing overall family functioning. The GF12 was modified by the authors in terms of item wording, scoring system, and translation. Participants’ responses were measured on a four-point Likert scale, ranging from “strongly disagree” (score = 1) to “strongly agree” (score = 4). Total scores ranged from 12 (indicating poor family functioning) to 48 (indicating optimal family functioning) [26].

### Tools validity and reliability

First, the authors modified the study tools and then translated them into Arabic. Following this, a panel of five specialists in community health nursing, psychiatric nursing, geriatric nursing, and nursing administration evaluated the face and content validity of the tools. Based on their feedback, the necessary adjustments were made. Additionally, the tools were assessed for internal consistency (reliability) using Cronbach’s alpha coefficient. The results were as follows: (1) The Multidimensional Emotional Abuse Questionnaire (MMEAQ) demonstrated good overall reliability (0.87), with subscales for restrictive engulfment, denigration, hostile withdrawal, and dominance/intimidation showing reliabilities of 0.93, 0.84, 0.83, and 0.87, respectively. (2) The Satisfaction with Life Scale (SWLS) had good reliability (0.81). (3) The General Functioning 12-item subscale (GF12) showed good reliability (0.84). (4) The Stress Scale had acceptable reliability (0.79). Finally, the authors conducted a confirmatory factor analysis for the four adapted tools to examine the relationships between the items and their corresponding variables. The results indicated that all four models had a good fit, as shown in Table 1. This preparatory phase lasted from mid-December 2024 to mid-February 2025.

**Table 1:** Summary of the confirmatory factor analysis for the four adapted tools

The MMEAQ (Two-factor model)			The GF12-item (Single-factor model)			The stress scale (Single-factor model)			The SWLS (Single-factor model)		
Factors	Factor loading	Fit indices	Factors	Factor loading	Fit indices	Factors	Factor loading	Fit indices	Factors	Factor loading	Fit indices
Restrictive engulfment	They ranged from 0.49 to 0.72	Root-Mean-Square Error of Approximation (RMSEA) = 0.047, Comparative Fit Index (CFI) = 0.963, Tucker-Lewis index (TLI) = 0.951, Goodness of Fit Index (GFI) = 0. 952, and χ2 = 744.46, DF = 344	Item 1	0.61	Root-Mean-Square Error of Approximation (RMSEA) = 0.034, Comparative Fit Index (CFI) = 0.963, Tucker-Lewis index (TLI) = 0.955, Goodness of Fit Index (GFI) = 0. 952, and χ2 = 1009.07, DF = 524	Item 1	0.73	Root-Mean-Square Error of Approximation (RMSEA) = 0.064, Comparative Fit Index (CFI) = 0.966, Tucker-Lewis index (TLI) = 0.952, Goodness of Fit Index (GFI) = 0. 957, and χ2 = 859.47, DF = 394	Item 1	0.75	Root-Mean-Square Error of Approximation (RMSEA) = 0.049, Comparative Fit Index (CFI) = 0.968, Tucker-Lewis index (TLI) = 0.959, Goodness of Fit Index (GFI) = 0. 957, and χ2 = 906.33, DF = 481
			Item 2	0.69		Item 2	0.61				
			Item 3	0.66		Item 3	0.78		Item 2	0.73	
Denigration	They ranged from 0.45 to 0.85		Item 4	0.72							
						Item 4	0.77				
			Item 5	0.70							
			Item 6	0.78		Item 5	0.85		Item 3	0.86	
			Item 7	0.56							
						Item 6	0.54		Item 4	0.65	
			Item 8	0.80							
Hostile withdrawal	They ranged from 0.56 to 0.84		Item 9	0.63		Item 7	0.72		Item 5	0.74	
			Item 10	0.73							
			Item 11	0.79							
Dominance	They ranged from 0.53 to 0.81		Item 12	0.84							

### Pilot study

Before data collection, a pilot study was conducted with five elderly couples experiencing spousal emotional abuse, representing 10% of the study sample. The pilot study aimed to verify and validate the feasibility, applicability, clarity, and relevance of the tools, as well as to assess the time required for participants to complete the questionnaires. The pilot sample was included in the main study sample since no modifications were made following the analysis of the pilot study results. This stage took place from mid-February 2025 until its conclusion.

### Ethical approval and consent to participate

Ethical approval for this study was granted by the Research Ethics Committee (code number 997) at the Faculty of Nursing, Menoufia University, Egypt, in accordance with the Declaration of Helsinki [27]. Formal approval was also obtained from the rural health unit of the respective villages to facilitate home visits and identify elderly couples experiencing spousal emotional abuse. Due to the sensitive nature of the personal data, informed consent was required prior to the initiation of the rehabilitative nursing intervention. Initially, the authors obtained informed consent from all study participants (*n* = 100). However, ninety other potential participants who were victims of spousal emotional abuse declined to participate, and were therefore excluded before the intervention began (Fig. 2). The purpose of the study was explained to the participants, and the authors assured them that the data collected would be used solely for the purposes of this study, with no associated risks. Confidentiality of the data was guaranteed. Participants were informed that their involvement in the study was entirely voluntary, and they could withdraw at any time without facing any penalties.

### Fieldwork

The fieldwork for this study took place from the beginning of March 2025 to the end of September 2025, lasting a total of seven months. The program was implemented in five phases:

#### Phase I (Relationship Building)

After receiving formal approval to conduct the study, the researchers introduced themselves to the elderly couples experiencing spousal emotional abuse. They provided a brief explanation of the study’s purpose, benefits, and timeline. The researchers met with the participants at their homes at times that were convenient for them.

#### Phase II (Assessment)

This phase lasted for one month, from the beginning of March 2025 until the end of the month. During home visits, the researchers distributed the study tools to 50 elderly couples who met the screening question and inclusion requirements, while avoiding the exclusion criteria. These tools were used to assess the couples’ experiences of emotional abuse, overall family functioning, life satisfaction, and stress levels before the rehabilitative nursing intervention was applied. The study tools were then individually assembled and coded by the researchers. The data collected during this phase served as baseline data and were analyzed to assess the needs of the 50 elderly couples in relation to spousal emotional abuse. Each participant took approximately 30 to 35 min to complete the tools.

#### Phase III (Planning)

This phase lasted for one month, from the beginning of April 2025 until the end. Based on the results of the assessment phase and a comprehensive review of relevant literature, the researchers designed the rehabilitative nursing intervention. During this phase, the overall goal of the intervention was established. The intervention aimed to improve the elderly couples’ practices concerning spousal emotional abuse, as outlined in Fig. 1. The appropriate teaching methods and media were selected, and a schedule for the home visits to conduct the intervention sessions was arranged. Additionally, the researchers developed the intervention content, which addressed various aspects of emotional abuse, as detailed in Table 2.

**Table 2:** Content and sessions of the rehabilitative nursing intervention

No. of Sessions	Aim	Content
1	• To build a professional relationship with the elderly couples. • To provide essential knowledge about emotional abuse.	• Introduction to the rehabilitative nursing intervention • Overview of emotional abuse: types, risk factors, manifestations, process, and consequences
2	• To establish therapeutic cooperation and ensure the elderly couples feel supported and understood. • To improve communication between the elderly couples.	• Sharing relationship, conflict, and marriage history • Identifying problematic communication patterns • Scenario discussion: Conflict resolution in a hypothetical relationship
3	• To help the elderly couples replace abusive behaviors with healthier ones.	• Violence as an initial step toward solving the emotionally abusive relationship • Development of emotional intelligence • Safe anger management techniques • Sharing authority and control in a relationship • Self-control and mood enhancement
4	• To strengthen interactions and promote secure attachment between the elderly couples.	• Promoting the expression of feelings • Building self-esteem through positive conversations • Development of autonomy
5	• To boost self-confidence and emotional health, and help manage stress among elderly couples.	• Techniques for self-orientation reconstruction • Decision-making strategies • Problem-solving and coping strategies • Stress management techniques, including meditation and deep breathing exercises • Relaxation techniques for better sleep
6	• To assist the elderly couples in overcoming the consequences of emotional abuse and reclaiming their lives.	• Teaching self-care practices • Establishing healthy boundaries • Management of self-blame • Establishment of support system
7	• To enhance family relationships and improve life satisfaction among elderly couples.	• Different roles in the family • Creation of a happy stable home environment • The “change-no-change-back” cycle • Improvement of social relations • Promotion of couple acceptance
8	• To enhance physical and mental well-being of elderly couples.	• Empowerment of self-care, lifestyle, and nutritional needs • Educating on the dangers of smoking and alcohol consumption • Increasing emotional self-awareness and partner-awareness

#### Phase IV (Implementation)

This phase lasted for two months, from May to June 2025. During this time, eight counseling sessions were delivered to the elderly couples through home visits, once a week, as part of the intervention. Each session lasted 60 min and was conducted during the daytime. In total, the rehabilitative nursing intervention spanned 8 h. Additionally, the researchers provided the elderly couples with a rehabilitative intervention booklet, written in simple Arabic, to serve as a reference for future use. The researchers began the rehabilitative nursing intervention by orienting the elderly couples to its goals, importance, and content. Before each session, the researcher provided an introduction, outlining the session’s objectives, followed by a brief summary of the previous session. Each session concluded with a summary, and the researcher addressed any questions or concerns from the elderly couples. During the implementation phase, various assistive media, including educational videos, posters, and podcasts, were used. In addition, a range of learning strategies, such as lectures, brainstorming, and group activities, were employed. Different evaluation methods, including pre- and post-session tests and feedback, were also implemented.

#### Phase V (Evaluation)

This phase lasted for one month, through September 2025. During the evaluation phase, post-intervention data were collected using the same study tools to assess the effectiveness of the rehabilitative nursing intervention on the elderly couples experiencing spousal emotional abuse. The data collection procedure followed the same process as in the assessment phase.

### Statistical analysis

Version 25 of the Statistical Package for Social Sciences (SPSS) was used to manage the data (IBM Corp., Armonk, NY, USA). The normality of the data distribution was assessed using the Kolmogorov-Smirnov test. Descriptive statistics for quantitative data were presented as means and standard deviations, while categorical data were expressed as frequencies and percentages. The mean scores of the quantitative variables were compared between intervention phases using the paired sample t-test. Cronbach’s alpha coefficient was used to assess the reliability of the study tools. Additionally, confirmatory factor analysis was performed to examine the relationships between the items and their corresponding variables. Statistical significance was set at *P* < 0.05.

## Results

**Table 3:** Frequency distribution of the study sample according to their personal characteristics (*n* = 100)

Personal characteristics	No.	%
**Age**		
60–70 years	86	86.0
>70 years	14	14.0
Mean ± SD	65.8 ± 4.7 years	
**Job**		
Worked	56	56.0
Not worked	44	44.0
**Education** 1ry education	59	59.0
2ry education	41	41.0
**Chronic diseases**		
Yes	57	57.0
No	43	43.0
**Family income**		
Sufficient	65	65.0
Not sufficient	35	35.0
**Smoking**		
Yes	40	40.0
No	60	60.0
**Alcohol**		
No	100	100.0
**Marijuana**		
No	100	100.0
**Marriage duration**	42.1 ± 6.5 years	
**Wetness abuse by parents**		
Yes	33	33.0
No	67	67.0

Table 3 demonstrates that approximately 86% of the elderly couples were aged between 60 and 70 years, with a mean age of 65.8 ± 4.7 years. The mean marriage duration was 42.1 ± 6.5 years. More than one-third (40%) of the couples were smokers, while 59% had primary education. About one-third (33%) of the elderly couples had previously witnessed emotional abuse between their parents. Additionally, approximately 65% of the couples reported having sufficient income.

**Table 4:** Comparing the elderly couples’ emotional abuse between the intervention phases (*n* = 100)

Emotional abuse dimensions	Pre-intervention		Post-intervention		Paired t test	
	Mean	± SD	Mean	± SD	t	*p*-value
Restrictive engulfment	28.5	7.9	19.5	6.7	24.8	< 0.001*
Denigration	30.7	6.9	23.2	6.1	15.7	< 0.001*
Hostile withdrawal	28.6	7.7	21.1	7.4	12.9	< 0.001*
Dominance/intimidation	29.9	4.5	21.5	5.2	20.1	< 0.001*
*Total emotional abuse*	117.7	21.4	85.2	20.3	30.5	< 0.001*

(*) Statistically significant at *p* < 0.05

Table 4 shows that the post-intervention mean scores for all dimensions of emotional abuse significantly decreased compared to the pre-intervention scores, with p-values < 0.05. Overall, the total emotional abuse score for the elderly couples significantly decreased in the post-intervention phase (85.2 ± 20.3) compared to the pre-intervention phase (117.7 ± 21.4), with a p-value < 0.05.

**Table 5:** Comparing the elderly couples’ stress scores between the intervention phases (*n* = 100)

Stress scale items	Pre-intervention		Post-intervention		Paired t test	
	Mean	± SD	Mean	± SD	t	*p*-value
Found it hard to wind down	2.64	0.482	1.33	0.372	23.26	< 0.001*
Tended to over-react to situations	2.51	0.502	1.10	0.346	17.51	< 0.001*
Using a lot of nervous energy	1.90	0.689	0.89	0.209	16.52	< 0.001*
Getting agitated	2.07	0.867	0.70	0.258	26.08	< 0.001*
Difficult to relax	2.50	0.502	0.87	0.338	24.09	< 0.001*
Intolerant of anything kept me from getting on with what was doing	2.68	0.373	1.38	0.487	18.66	< 0.001*
Felt that I was rather touchy	1.71	0.456	0.54	0.114	22.78	< 0.001*
*Total stress scale*	16.2 ± 3.1		6.7 ± 1.9		65.5	< 0.001*

(*) Statistically significant at *p* < 0.05

Table 5 reveals that the post-intervention mean scores for all items of the stress scale significantly decreased compared to the pre-intervention scores, with p-values < 0.05. Overall, the total stress score for the elderly couples significantly decreased in the post-intervention phase (6.7 ± 1.9) compared to the pre-intervention phase (16.2 ± 3.1), with a p-value < 0.05.

**Fig. 3:**
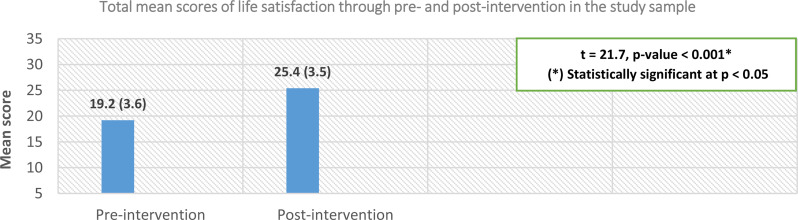
Comparing the elderly couples’ life satisfaction between the intervention phases (*n* = 100)

Figure 3 demonstrates that the elderly couples’ mean scores for total life satisfaction significantly increased in the post-intervention phase (25.4 ± 3.5) compared to the pre-intervention phase (19.2 ± 3.6), with a p-value < 0.05.

**Fig. 4:**
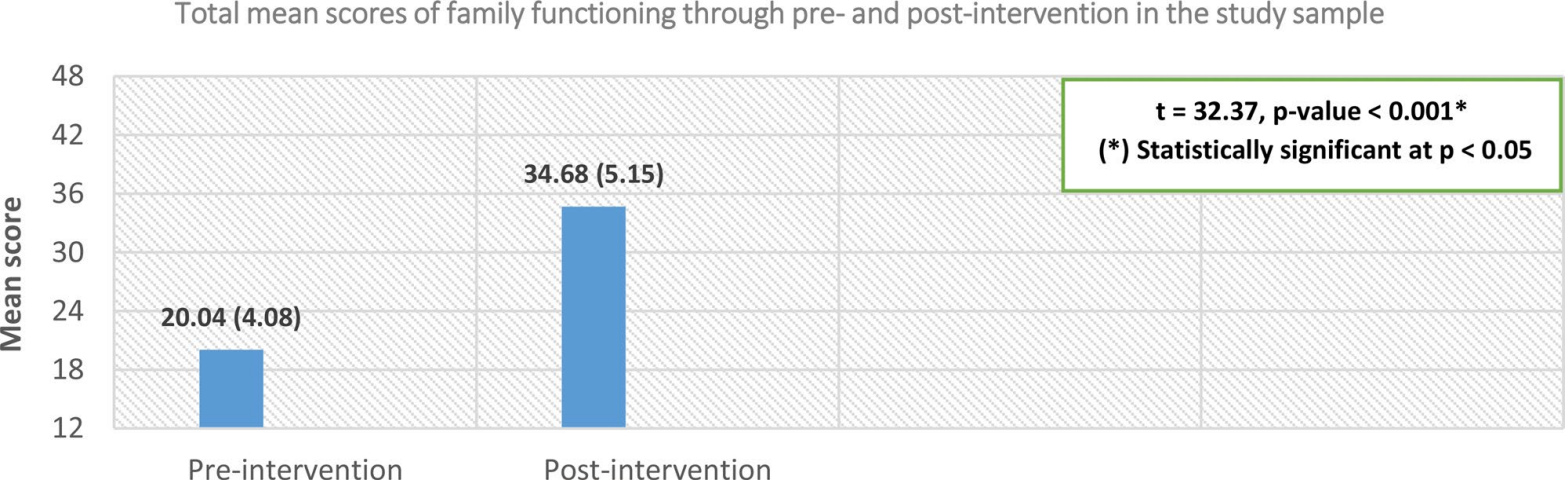
Comparing the elderly couples’ family functioning between the intervention phases (*n* = 100)

Figure 4 shows that the elderly couples’ mean scores for total family functioning significantly increased in the post-intervention phase (34.68 ± 5.15) compared to the pre-intervention phase (20.04 ± 4.08), with a p-value < 0.05. This result, along with the findings in Tables 4 and 5; Fig. 4, supports the hypothesis of this study, which stated: “Elderly victims of spousal emotional abuse who receive the rehabilitative nursing intervention will show a reduction in stress levels and an improvement in both life satisfaction and family functioning,” as illustrated in Fig. 1.

## Discussion

Elder abuse is a serious public health concern and a top priority in addressing aging-related issues [28]. The World Health Organization reports that approximately one in six elderly individuals worldwide has experienced some form of maltreatment [29]. Spousal emotional abuse, a form of intimate partner violence, can have significant negative consequences on both individual well-being and relationship outcomes [18]. Elderly individuals, in particular, are vulnerable to domestic violence [20]. Therefore, the present study aimed to evaluate the impact of a rehabilitative nursing intervention for elderly victims of spousal emotional abuse on stress level, life satisfaction, and family functioning.

**Regarding the impact of the rehabilitative nursing intervention on emotional abuse among elderly couples**, the present study found that the post-intervention scores for all dimensions of emotional abuse, as well as total emotional abuse, significantly decreased compared to the pre-intervention scores. These findings support the study hypothesis. These statistically significant decreases can be attributed to the empowerment of elderly couples through effective training and the rehabilitative nursing intervention, which aimed to enhance their responses to spousal emotional abuse. The intervention provided the couples with techniques and strategies for managing emotional abuse, including emotional intelligence, anger management, sharing authority and control in relationships, self-control, mood enhancement, expressing feelings, developing self-esteem and autonomy, and managing self-blame. Additionally, the intervention booklet included practical scenarios related to emotional abuse. Furthermore, the elderly couples were highly motivated to participate in the study, and the intervention was tailored based on the results of the assessment phase.

The current results align with those of a study conducted by Hazrati et al. [20] in Iran, which assessed the effect of an emotion-focused intervention on spousal emotional abuse and marital satisfaction. The findings indicated that abusive behaviors among elderly couples in the experimental group significantly decreased after eight sessions of the intervention. Another study conducted by Panabad et al. [30] in Iran evaluated the effectiveness of emotion-focused therapy in reducing marital violence and improving marital adjustment. The results showed statistically significant decreases in marital psychological violence among the intervention group after the counseling sessions. Similarly, the findings are consistent with those of a study by El-Amrosy et al. [18] in Egypt, which assessed emotion-focused, couple-based interventions for wives’ emotional abuse and marital satisfaction. The results showed significant improvements in emotional abuse after nine intervention sessions.

**Regarding the impact of the rehabilitative nursing intervention on stress among the elderly couples**, the present study found that the post-intervention scores for all stress items, as well as total stress, significantly decreased compared to the pre-intervention scores. These findings support the study hypothesis. The significant reductions in stress can be attributed to the positive effects of the intervention. First, the intervention helped the elderly couples reduce efforts to monitor their partners’ whereabouts and isolate them from social interactions or self-enhancing activities, which led to improved relaxation. Second, it prevented the couples from putting each other down through insults, criticism, or belittling, thus boosting their confidence. Third, the intervention introduced various strategies—such as problem-solving, coping strategies, meditation, deep breathing exercises, and relaxation techniques—that positively impacted their stress levels. These results align with those of a study by Evandrou et al. [31] in India, which examined the association between elder abuse and psychological distress. Their findings showed that elder abuse victims experienced significantly higher levels of stress compared to non-victims. Similarly, a study by Zhang et al. [32] in the USA assessed the link between psychological distress, abusive experiences, and help-seeking behaviors, revealing significant differences in psychological distress between victims and non-victims of intimate partner violence.

**Regarding the impact of the rehabilitative nursing intervention on life satisfaction among the elderly couples**, the present study found a significant increase in life satisfaction post-intervention compared to pre-intervention scores, supporting the study hypothesis. The statistically significant improvements can be attributed to the positive effects of the intervention. First, the intervention helped the elderly couples avoid using threats, coercion, or other intimidating behaviors to control their partners, which enhanced their sense of freedom and autonomy. Second, it provided them with various strategies that positively influenced their life satisfaction and overall well-being. These strategies included creating a happy and stable home environment, fostering couple acceptance, managing self-blame, and establishing a support system. The current findings align with those of El-Amrosy et al. [18], whose study showed a statistically significant improvement in life satisfaction following emotion-focused couple-based interventions. Similarly, studies by Hazrati et al. [20] and Dinmohammadi et al. [33] revealed that life satisfaction among elderly couples in the experimental group significantly increased after eight sessions of emotion-focused intervention.

**Regarding the impact of the rehabilitative nursing intervention on family functioning among the elderly couples**, the present study revealed a significant improvement in family functioning post-intervention compared to pre-intervention scores, supporting the study hypothesis. These statistically significant improvements can be attributed to the positive effects of the rehabilitative nursing intervention. First, the intervention helped elderly couples avoid emotional and psychological withdrawal, which is often used as a form of control or punishment. This contributed to better communication, more open discussions, and clearer role definitions between partners. Second, the intervention provided the elderly couples with various strategies to improve family functioning, such as understanding family roles and effective conflict resolution. These results align with those of a study by Anousheh et al. [34] in Iran, which found that family functioning significantly improved after emotion-focused therapy sessions in the intervention group. Similarly, a study by Cong et al. [25] in Malaysia reported that short-term emotional counseling significantly enhanced family functioning among Malaysian couples experiencing emotional violence.

### Limitations

Despite the positive findings, there are several limitations to consider. First, the use of a purposive sampling technique may limit the generalizability of the results because of a lack of representation. The strict inclusion and exclusion criteria applied in this study could affect the broader applicability of the findings. Additionally, since the data were self-reported, there is potential for response bias, which may influence the extent to which these results can be generalized.

## Conclusion

Statistically significant reductions were observed in the elderly couples’ emotional abuse and stress levels, while their life satisfaction and family functioning significantly improved in the post-intervention phase compared to the pre-intervention phase. Based on these results, it can be concluded that the rehabilitative nursing intervention for elderly victims of spousal emotional abuse effectively reduced their stress levels and improved their life satisfaction and family functioning, thereby supporting the research hypothesis.

### Implications for practice

The nursing rehabilitative intervention has proven effective in reducing spousal emotional abuse and stress levels, while also improving life satisfaction and family functioning among elderly couples. Based on the study’s findings, the following recommendations are proposed: seek support from specialized professionals and institutions to implement the nursing rehabilitative intervention and prioritize self-care for elderly couples; strengthen the role of rural health units in addressing spousal emotional abuse by establishing dedicated psychological support units; conduct regular surveys by community institutions to assess the prevalence and severity of emotional abuse among elderly couples; launch community initiatives and social campaigns focused on managing emotional abuse, especially among elderly couples; and utilize television and other media to deliver emotional counseling interventions, helping to alleviate emotional and psychological conflicts within elderly couples.

## Data Availability

The materials and data used in this study are not available to the public due to confidentiality considerations. They are available from the corresponding author upon reasonable request.

## Abbreviations

MMEAQ: Multidimensional Emotional Abuse Questionnaire
DASS-21: Depression, Anxiety, and Stress Scale-21 Items
SWLS: Satisfaction with Life Scale
GF12: General Functioning 12-item subscale
FAD: McMaster Family Assessment Device
